# Bronchopleural Fistula and Endobronchial Valve Placement in a Patient With COVID-19 Pneumonia: A Case Report With Literature Review

**DOI:** 10.7759/cureus.24202

**Published:** 2022-04-17

**Authors:** Zaryab Umar, Usman Ilyas, Salman Ashfaq, Deesha Shah, Mahmoud Nassar, Theo Trandafirescu

**Affiliations:** 1 Internal Medicine, Icahn School of Medicine at Mount Sinai, Queens Hospital Center, New York, USA; 2 Internal Medicine, Allama Iqbal Medical College, Lahore, PAK; 3 Medicine, Icahn School of Medicine at Mount Sinai, New York, USA

**Keywords:** interventional pulmonology, pneumothorax, covid-19, endobronchial valve, bronchopleural fistula

## Abstract

Bronchopleural fistulas (BPFs) are associated with high morbidity and mortality. Though most commonly seen after surgical interventions, they are increasingly reported as complications of COVID-19 infection. We present the case of an 86-year-old man with COVID-19 pneumonia and subsequent bronchopleural fistula (BPF) with persistent air leak. Endobronchial valves were placed in apical and posterior segments of the right upper lobe resulting in successful cessation of the air leak. The purpose of the case report and literature review is to help guide the management of persistent air leak.

## Introduction

A bronchopleural fistula (BPF) is a communication between the bronchial tree and the pleura, allowing air to leak into the pleural space, resulting in high morbidity and mortality [1]. The most common cause of BPF is surgical procedures with an incidence of 4.5-20% after pneumonectomy and 0.5% after lobectomy [2]. BPF can also be seen as a consequence of chronic obstructive pulmonary disease (COPD), necrotizing lung infections, radiotherapy, and chemotherapy [3]. Recently, BPFs have been recognized as a rare but serious complication of SARS-CoV-2 pneumonia. We present the case of an 86-year-old male with COVID-19 pneumonia who developed right-sided pneumothorax as a result of right upper lobe BPF requiring endobronchial valve (EBV) placement.

## Case presentation

**Figure 1:**
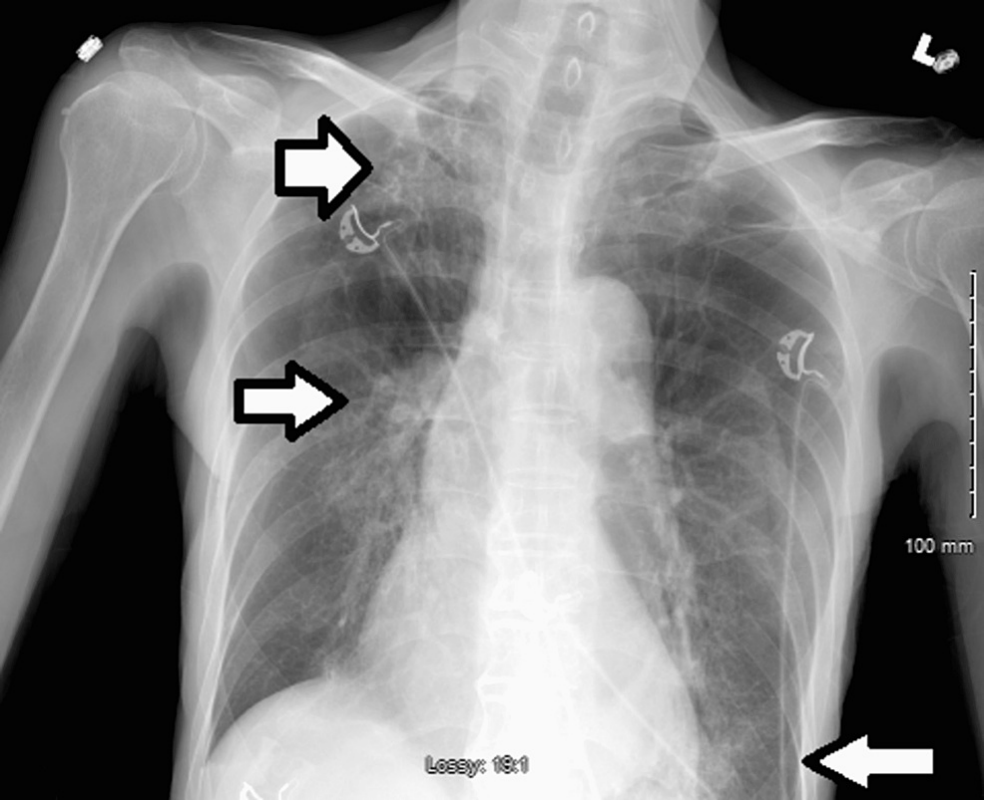
Chest X-ray at the presentation showing hyperinflated lung, patchy reticular multifocal opacities in the right apex, right hilum, and left base (white arrows).

An 86-year-old Southeast Asian male with a past medical history of hyperlipidemia and COPD presented to the emergency department accompanied by his family with complaints of worsening shortness of breath that began three days ago. The patient reported that his shortness of breath is worse at night, persistent, and associated with a dry cough. There are no other aggravating or relieving factors. Six months prior to the hospital presentation, the patient received the Johnson and Johnson COVID-19 vaccine. On examination, the patient was found to be in respiratory distress, tachypneic, hypoxic to 84% oxygen saturation on room air which was improved to 95% with 4 liters of nasal cannula (NC), blood pressure of 101/60 and bilateral lower lung base rhonchi on auscultation. The polymerase chain reaction test for COVID-19 infection was positive. Initial laboratory results revealed leukocytopenia, lymphocytopenia, neutrophilia, bandemia, respiratory alkalosis, and mild hyponatremia. Troponin and pro-BNP remained within normal limits and EKG showed normal sinus rhythm without acute changes. Procalcitonin was elevated to 5.70 ng/ml, as were inflammatory markers including C-reactive protein (CRP), lactate dehydrogenase (LDH), and ferritin, while D-dimer remained within age-appropriate normal limits (Table 1). A chest X-ray (CXR) revealed hyperinflated lungs, patchy reticular multifocal opacities in the right apex, right hilum, and left base (Figure 1).

**Table 1:** Lab values obtained at the time of patient’s initial presentation.

Labs obtained at the time of presentation	Lab value	Normal range and reference units
White blood cell count	3.53	4.80-10.80 x 10^3^/mcL
Lymphocyte%	5.9	20.0-45.0%
Neutrophil%	89.0	44.0-70.0%
Bands manual%	27.0	0.0-5.0%
Sodium	135	136-145 mmol/L
PH venous	7.42	7.32-7.43
PCO2 venous	37	41-54 mmHg
Bicarbonate venous	24	22-29 mmol/L
Procalcitonin	5.70	0.02-0.10 ng/mL
C-Reactive Protein (CRP)	108.40	0.02-0.10 ng/mL
Lactate dehydrogenase (LDH)	334	135-225 U/L
Ferritin	1123	300-400 ng/mL
D-dimer	220	0-243 ng/mL DDU

The patient was admitted to the medicine floor and started on Remdesivir (200 mg IV loading dose followed by 100 mg IV daily), dexamethasone (10 mg daily instead of 6 mg given the patient’s respiratory status), and Azithromycin (500 mg IV daily) with an initial oxygen saturation of 95% on 4 liters NC. He also received a one-time dose of 400 mg of Tocilizumab in the emergency department. The patient continued to have increasing oxygen requirements and was put on 60 liters high flow nasal cannula (HFNC) with a FiO2 of 90%. The patient was saturating at 94%. This was later weaned to 6 liters NC as the patient’s oxygen requirement decreased with improvement in the clinical picture.

**Figure 2:**
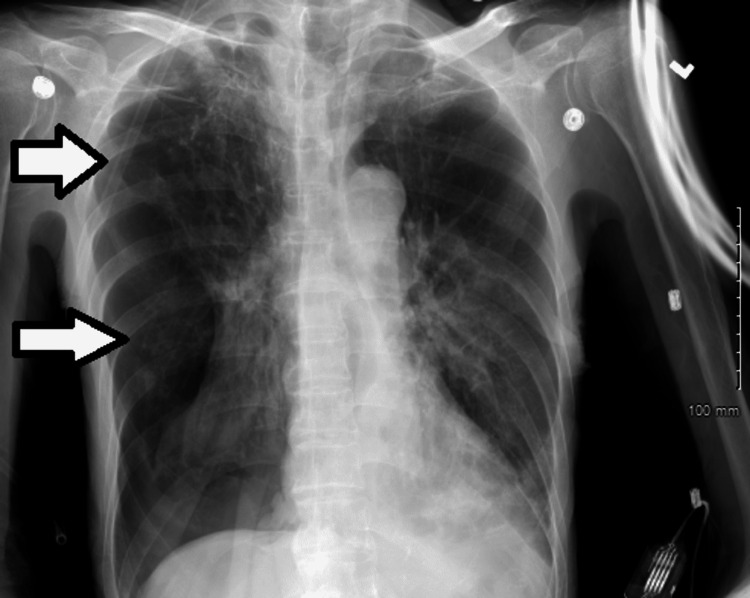
Chest X-ray showing lucency in the mid and lower right lung fields suggesting pneumothorax (white arrows).

On day 20 of admission, the patient was in respiratory distress requiring 60 liters of HFNC and a FiO2 of 70% with absence of breath sounds on the right hemithorax. CXR revealed a large right-sided pneumothorax requiring immediate chest tube insertion (Figure 2).

Serial CXRs were performed which revealed a gradual decrease in the size of the pneumothorax in conjunction with continuous chest tube suctioning maintained at -20 cm H2O. The patient's respiratory status gradually improved, and the oxygen requirement was eventually titrated down to a 2-liter nasal cannula with 96% oxygen saturation.

**Figure 3:**
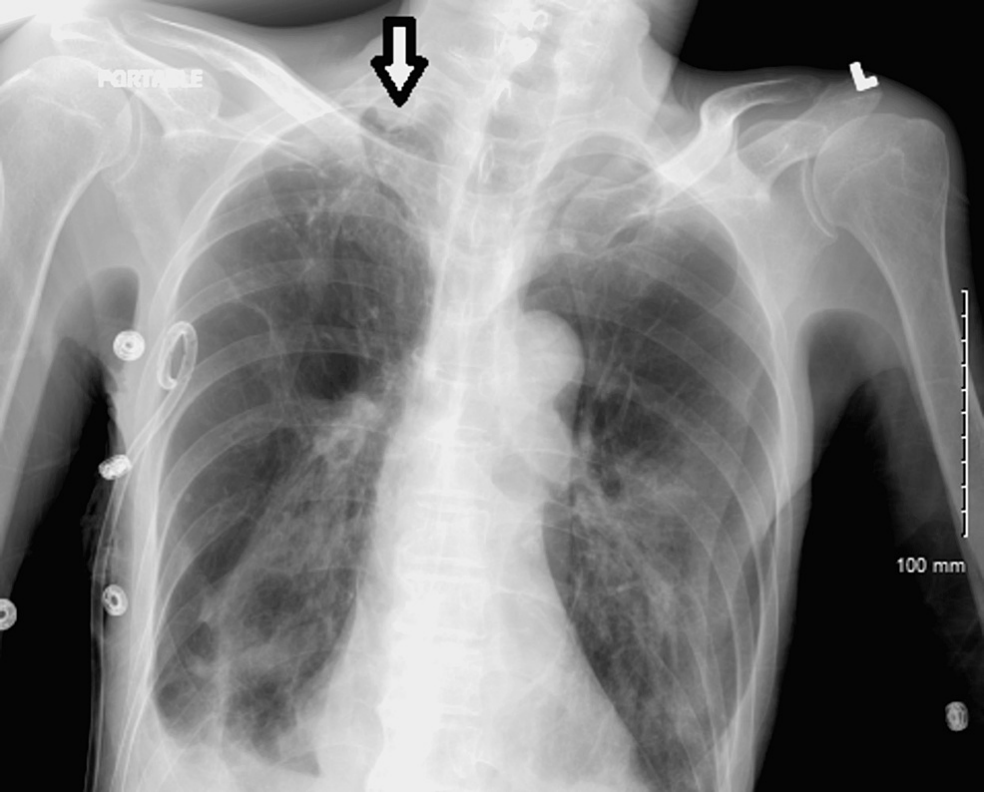
The chest X-ray indicates a small right apical pneumothorax (white arrow).

**Figure 4:**
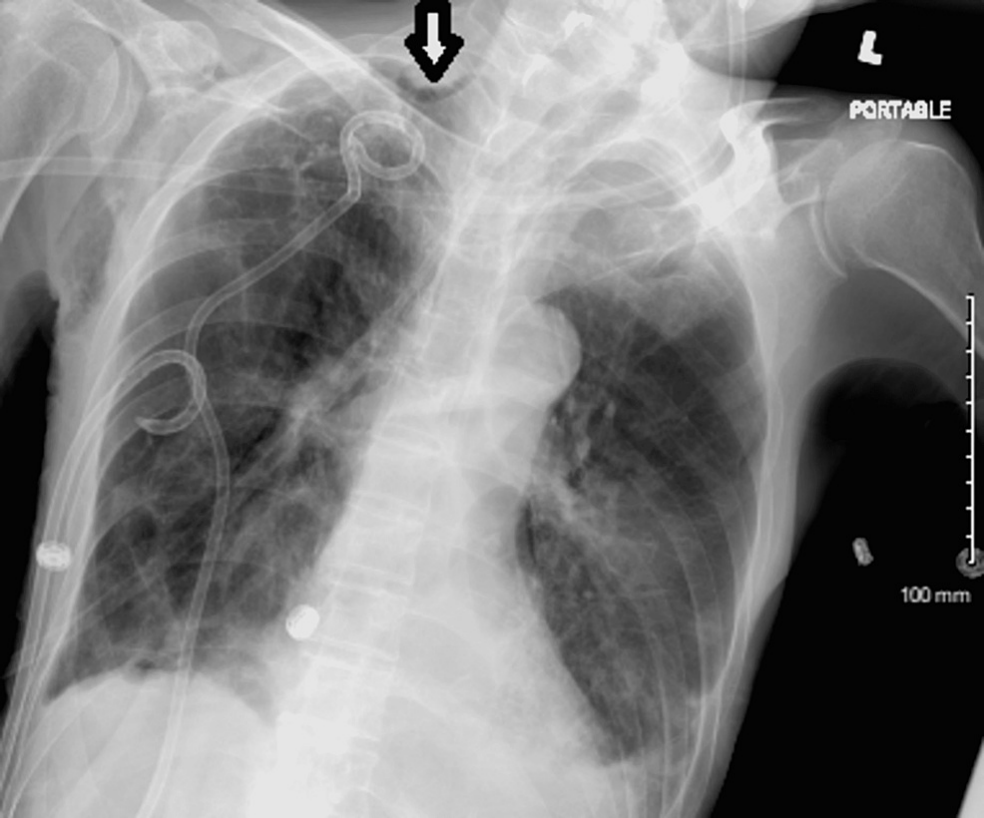
Follow-up chest X-ray after apical chest tube insertion: No evidence of gross pneumothorax, but a stable small right apical pneumothorax can be appreciated (white arrow).

On the 24th day after admission, serial CXRs revealed the development of a right apical pneumothorax, which led to the placement of a right apical chest tube (Figure 3). Follow-up CXR showed no evidence of gross pneumothorax (Figure 4).

**Figure 5:**
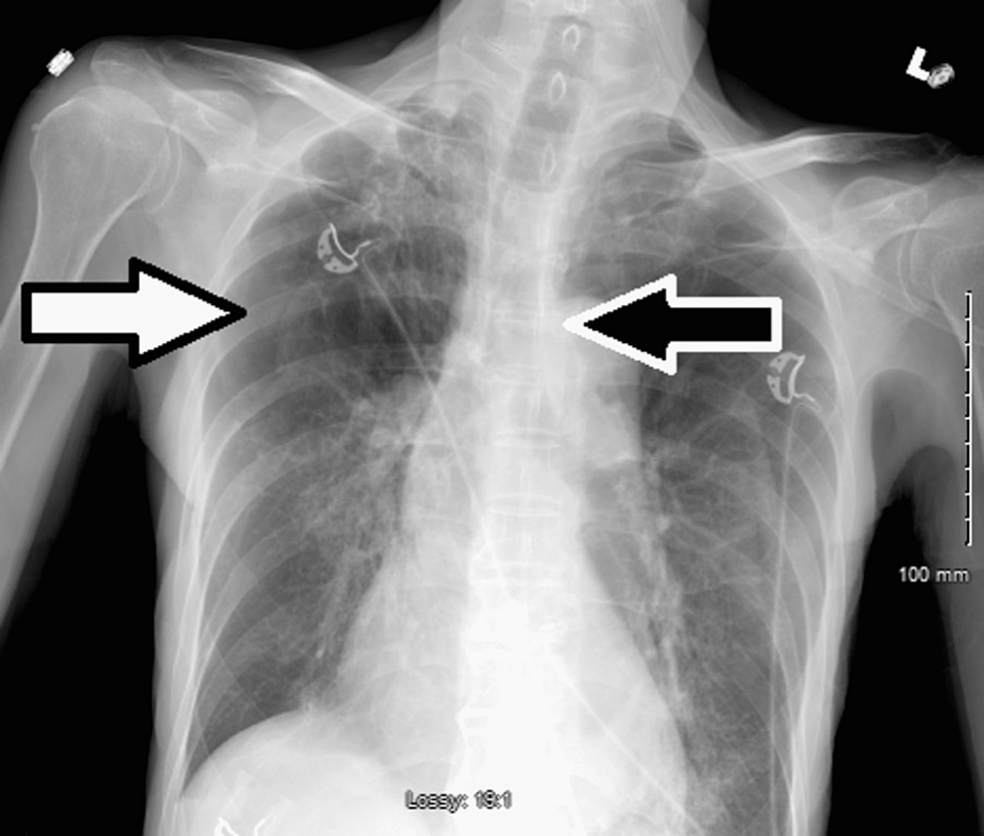
Chest X-ray obtained after apical chest tube removal, large right-sided pneumothorax (white arrow) with flattening of the right mediastinal structures and mediastinal shift to the left (black arrow). Findings suggestive of tension pneumothorax.

**Figure 6:**
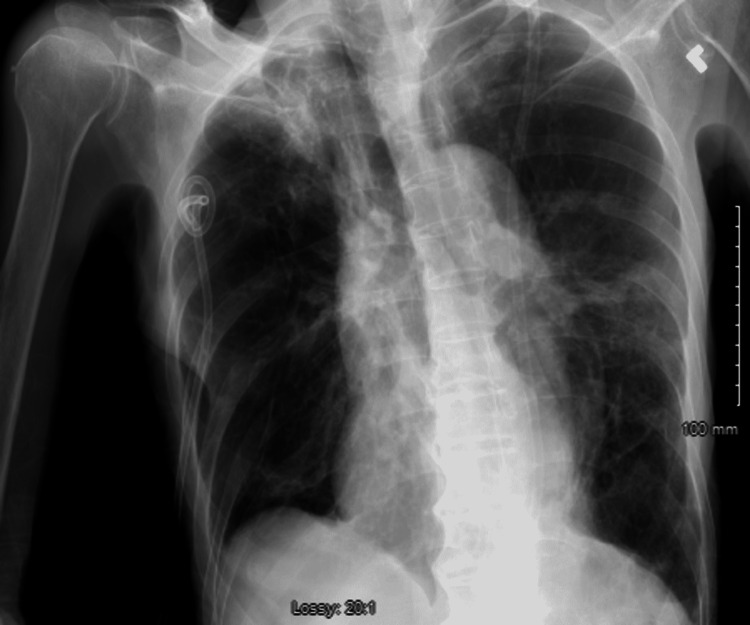
Chest X-ray obtained after reinsertion of the apical chest tube showed no large pneumothorax and resolution of the tension pneumothorax.

**Figure 7:**
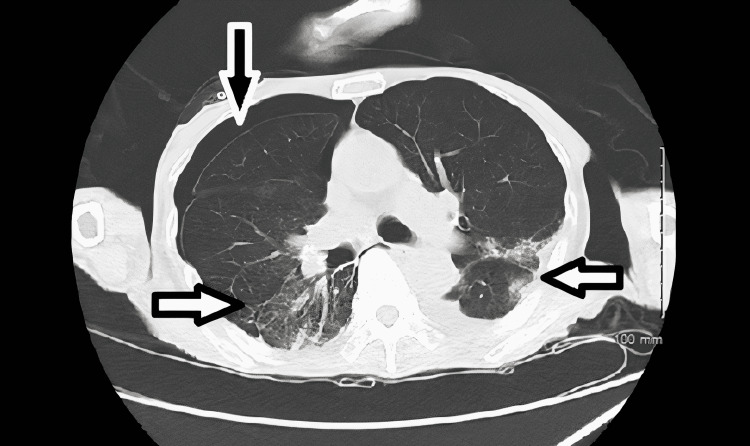
CT scan of the chest without intravenous contrast obtained prior to patient's transfer to another acute care facility showed moderate right-sided pneumothorax (black arrow) with bilateral ground-glass opacities indicative of infection/pneumonia (white arrows).

Clinical improvement and the absence of gross pneumothorax on imaging led to the decision to remove the chest tubes. After successful removal of the inferior chest tube, removal of the apical chest tube resulted in the development of tension pneumothorax (Figure 5). Emergent insertion of the apical chest tube was done with subsequent resolution of the tension pneumothorax (Figure 6). Follow-up chest X-rays suggested a significant improvement but not complete resolution of the apical pneumothorax. While the patient was hospitalized, attempts were made to wean him off of suction and remove the chest tube but were unsuccessful. CT scan of the chest revealed moderate right-sided pneumothorax with ground glass opacities suggestive of an inflammatory process (Figure 7). Eventually, a decision was made to investigate non-surgical definitive treatment options for which the patient was transferred to another hospital with a higher level of care due to a lack of resources at our hospital.

**Figure 8:**
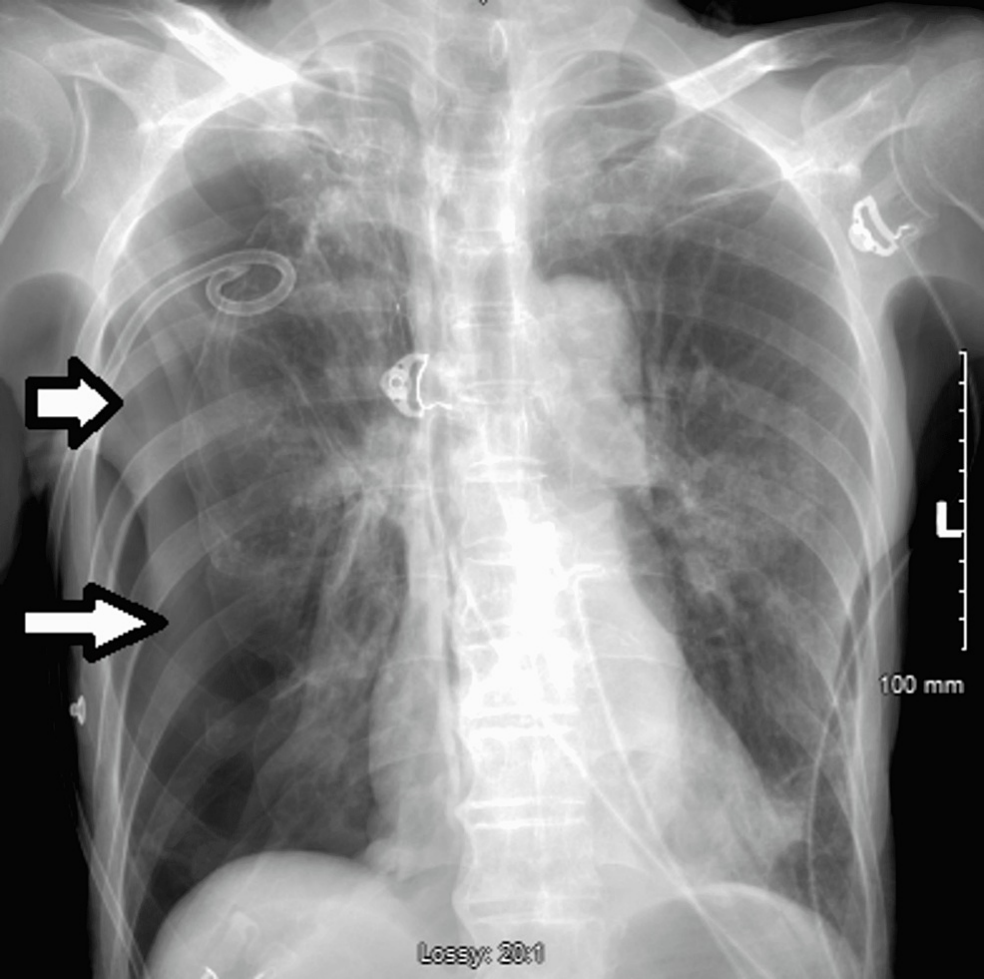
Chest X-ray suggestive of marked right-sided pneumothorax (white arrows).

The patient underwent endobronchial apical valve and blood patch placement to the apical segment of the right upper lobe without complications. CXR post-procedure day two revealed worsening pneumothorax without changes in respiratory status or oxygenation, and a chest tube was then placed to suction for one minute and later to water seal drainage (Figure 8).

**Figure 9:**
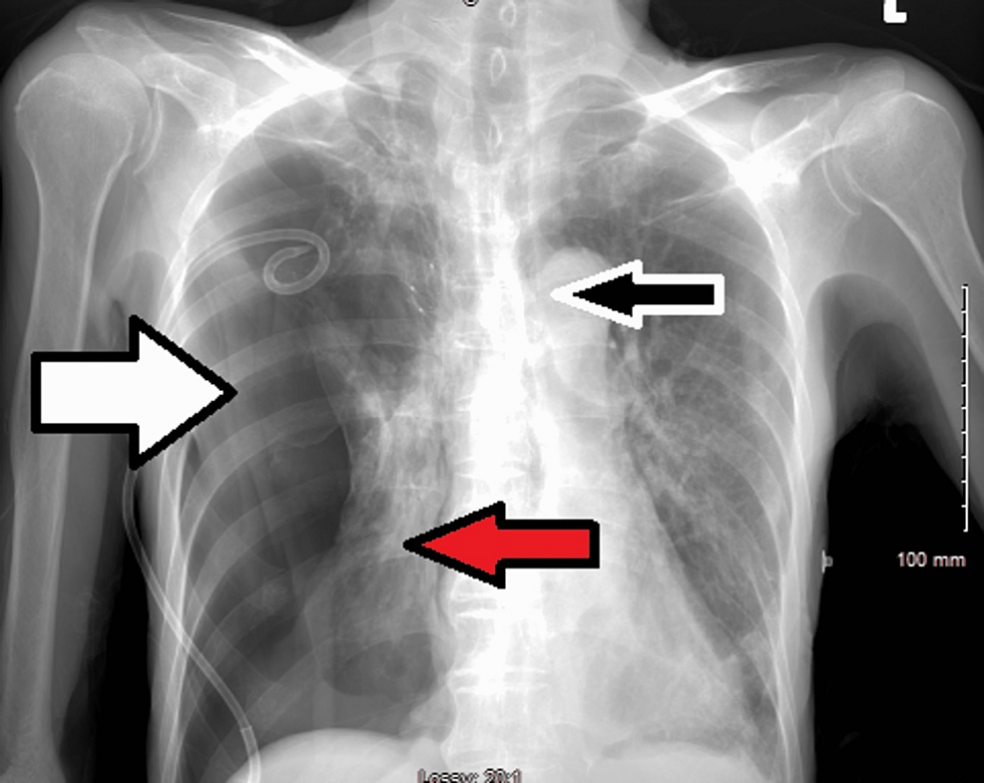
Chest X-ray showed pneumothorax (white arrow) with worsening atelectasis in the right lung (red arrow) and mild leftward shift in the midline (black arrow).

Ten days after the procedure, the patient continued to experience an air leak and lung expansion while under suction. Initially, it was believed that this would occur during the healing process of the bronchopleural fistula; however, additional valve placement was thought to be beneficial. Consequently, the patient underwent two additional endobronchial valve placements in the apical and posterior segments of the right upper lobe. A tension pneumothorax developed three days after the additional valves were placed, however, the patient remained hemodynamically stable (Figure 9). After removing the old chest tube, a new pigtail catheter was inserted, and suction was applied at -40 cm H2O.

Follow-up CXR showed a dramatic reduction in pneumothorax size. In subsequent CXRs, the pneumothorax improved slightly without further evidence of air leak. Later, the pigtail catheter was attached to water seal drainage and was removed from suctioning. The patient remained asymptomatic and was discharged to a sub-acute rehabilitation facility with a Heimlich valve attached to the chest tube. After an outpatient follow-up, the chest tube was removed since there were no air leaks from the Heimlich valve, and bilateral breath sounds were present throughout both lung fields.

## Discussion

A bronchopleural fistula is a connection between the bronchial tree and the pleura that can result in air leakage into the pleural space, causing high morbidity and mortality [1]. BPFs are most associated with surgical procedures on the lungs but can also occur as a consequence of COPD, necrotizing infections of the lungs, radiation, and chemotherapy [3]. It is generally recognized that this complication occurs after surgical procedures such as lobectomy and pneumonectomy. It occurs between 4.5%-20% after pneumonectomy and 0.5% to 1% following lobectomy [2]. A high mortality rate is observed, with rates varying from 18% to 67% [4], and the most common cause of death is aspiration pneumonia, followed by acute respiratory distress syndrome (ARDS) and/or tension pneumothorax [5]. Identifying the clinical presentation and signs of BPF is extremely important, and prompt chest tube placement with suctioning can reduce morbidity and mortality in patients, which can be used to establish a bridge to surgical or endobronchial correction [5].

A contrast-enhanced CT scan of the chest is the imaging of choice in suspect cases of fistulas [3,6], while direct visual inspection via bronchoscopy remains the gold standard for diagnosis [3,5]. Additional investigations can be conducted using single-photon emission tomography (SPECT) and computed tomography bronchography (CTB) [3]. Treatment depends on the etiology and size of the fistula. Fistulae caused by surgical procedures and/or exceeding 8 mm in size may require surgical correction, while fistulae caused by other etiologies, such as necrotizing infections or chemical ingestions, may require endobronchial intervention [3].

Several cases of BPF have been reported following the development of severe disease in cases of SARS-CoV-2 infection. Although the pathophysiology of bronchopleural fistulas in patients with COVID-19 pneumonia is still unclear, cavitary lesions have been reported as a possible cause [7]. In addition, intra-alveolar bleeding, which results in additional parenchymal damage, can be related to cavitations in the lungs [8].

An endobronchial valve allows air to exit the pleura during expiration but does not allow air back into the pleural space during inspiration [9]. For the management of BPF that developed in the patient secondary to SARS-CoV-2 infection, endobronchial valve placement with blood patch was performed instead of conventional surgery, as surgical correction has a high risk of failure in the context of friable lung tissues caused by infections [1,6].

Talon et al. describe a 64-year-old man who required a chest tube thoracostomy due to recurrent right-sided empyema [10]. The CT scan revealed a right middle lobe bronchopleural fistula with a moderately sized loculated pneumothorax. The patient was subsequently transferred to a nursing home after pleur-evac to -20 cm H2O on wall suction. Interventional radiology was consulted for endobronchial valve placement as the air leak persisted. A 9-mm spiration valve system EBV was placed into the right middle lobe airway. The EBV was successfully placed on repeat bronchoscopy, and the patient was discharged to the nursing facility. Six weeks after the placement of the EBV, the air leak was resolved, and the chest tube was removed. The EBV was removed 13 weeks after its placement without complications [10].

Donatelli et al. described two cases of alveolar pleural fistulas and their respective management options [11]. The patient, a 67-year-old Caucasian male, presented with fever and respiratory failure and was admitted with COVID-19 pneumonia. Due to worsening respiratory failure, the patient got transferred to another facility where continuous pressure airway via a helmet and Tocilizumab was started. His condition deteriorated further and he was intubated. Following successful extubation, the patient developed septic shock as a result of worsening CXR and Klebsiella pneumoniae bacteremia. The patient was intubated again. Five days following mechanical ventilation, the patient developed tension hydropneumothorax on the right side, resulting in chest tube placement and decompression. The CT scan revealed a large pneumothorax on the right side accompanied by pleural effusions. Klebsiella pneumoniae and Enterococcus faecalis were identified from bronchoalveolar lavage and the patient was prescribed Ampicillin and Meropenem. Despite the fact that the patient was successfully weaned eight days later, the pneumothorax persisted. Flexible bronchoscopy identified the middle lobe as the source of the leak, and two valves were placed in the segmentary bronchi of the middle lobe to reduce the leak. After the valve placement, subsequent imaging studies showed a reduction in pneumothorax that led to the removal of the chest tube and both EBVs without complications or recurrence of the pneumothorax [11].

A 73-year-old Caucasian male presented with fever and severe acute hypoxic respiratory failure and was admitted for COVID-19 pneumonia. However, due to worsening respiratory status the patient required ICU level of care with mechanical ventilation. Tracheostomy was done 10 days after ICU admission. On day 15, the occurrence of right hydro-pneumothorax required insertion of the chest drainage tube. Klebsiella pneumoniae and Enterococcus faecalis were isolated from pleural fluid, and the patient was started on Ampicillin and Meropenem. Though the patient improved significantly, the pneumothorax persisted. Bronchoscopy revealed a right lower lobe alveolar pleural fistula necessitating the placement of three valves in the segmental bronchi of the right lower lobe. Air leak was significantly reduced, but resolution resulted after the placement of a valve in the apical portion of the right lower lobe. Subsequent imaging studies done after the valve placement showed improvement in pneumothorax, warranting removal of the chest tube and the EBVs with no complications and recurrence of the pneumothorax [11].

Saha et al. report a 42-year-old male experiencing fever, cough, rhinorrhea, sputum production, and shortness of breath for one week. The patient was exposed to a COVID-19 patient and had been self-quarantining at home. However, 2 hours before presenting to the ED the patient developed severe anterior left-sided chest pain after a bout of vigorous coughing. Past medical history was significant for a 40-pack per year smoking history. Vitals were suggestive of tachycardia and tachypnea with an oxygen saturation of 84% that corrected to 92% on 6 liters nasal cannula. Chest auscultation revealed absent breath sounds in the left hemithorax. Chest X-ray revealed a large left-sided pneumothorax necessitating the need for a chest drainage tube. An Air leak was noted, and hence the chest tube connected to wall suction. Rapid antigen tests for SARS-CoV-2 and reverse transcriptase-PCR were both positive. The patient improved over the course of seven days, but pneumothorax and subcutaneous emphysema worsened with attempts to manage the air leak with only water seal drainage. Two additional chest tubes were placed with no resolution of the pneumothorax. CT scan revealed persistence of pneumothorax with bilateral apical bullous disease attributable to the patient's history of smoking. The patient underwent bronchoscopy, and three valves (spiration valve system), all 9 mm, were deployed in the apicoposterior, anterior and lingular bronchus. The air leak was resolved, and pleural drainage catheters were removed. EBVs were removed a few weeks after their insertion, and subsequent imaging showed partial expansion of the left upper lobe with residual areas of atelectasis [12].

The aforementioned case reports provide useful insight into the complications that can arise as a result of COVID-19 infection, particularly pneumothorax and persistent air leak. The cases also provide a guide to the management of such complications using EBV placement (Table 2). In our case, the patient underwent EBV placement but continued to have persistent air leak. He then underwent two additional EBV placements which resulted in significant improvement in the size of the pneumothorax. Chest tube was removed upon subsequent follow-up and the patient was noted to have bilateral breath sounds and no air leak.

**Table 2:** A review of four cases of COVID-19 infection and persistent air leak. BPF: Bronchopleural fistula; EBV: Endobronchial valve.

Author	Age	Gender	COVID-19 infection (present/absent)	Initial presentation	Complication	Persistent air leak (present/absent)	Intervention	Outcome
Talon et al., 2021 [10]	64	Male	Present	Recurrent right-sided empyema	Right middle lobe (RML) BPF and moderate-sized loculated right pneumothorax	Present	2 EBVs placed in RML airway	Reduction in pneumothorax, no recurrence and eventual removal of EBV
Donatelli et al., 2021 [11]	67	M	Present	Fever and respiratory failure requiring intubation	Right-sided pneumothorax, alveolar-pleural fistula (APF) in RML	Present	EBV placed in apical portion of RML	Reduction in pneumothorax, no recurrence and eventual removal of EBV
	73	M	Present	Respiratory failure requiring intubation	Right-sided pneumothorax, APF in right lower lobe (RLL)	Present	EBV placed in apical portion of RLL	Reduction in pneumothorax, no recurrence and eventual removal of EBV
Saha et al., 2021 [12]	42	M	Present	Fever, cough, sputum production, shortness of breath and left-sided pneumothorax	Persistent air leak in apicoposterior, anterior and lingular bronchus	Present	EBVs placed in the apicoposterior, anterior and lingular bronchus	Resolution of air leak, partial expansion of the left upper lobe and eventual removal of EBV

## Conclusions

Severe complications can arise as a consequence of SARS-CoV-2 infections and its proposed management. We are yet unsure of the actual incidence of BPF as a complication of SARS-CoV-2 or its exact pathophysiology. Continued reporting of these entities will lead to increased incidence and ultimately develop guidelines for prompt identification and management of BPF.
